# Phe-140 Determines the Catalytic Efficiency of Arylacetonitrilase from *Alcaligenes faecalis*

**DOI:** 10.3390/ijms21217859

**Published:** 2020-10-23

**Authors:** Jung-Soo Kim, Sanjay K. S. Patel, Manish K. Tiwari, Chunfen Lai, Anurag Kumar, Young Sin Kim, Vipin Chandra Kalia, Jung-Kul Lee

**Affiliations:** 1 Department of Chemical Engineering, Konkuk University, Seoul 05029, Korea; junnie05@hanmail.net (J.-S.K.); sanjaykspatel@gmail.com (S.K.S.P.); manish.bme@gmail.com (M.K.T.); cfenlai01@gmail.com (C.L.); anuragbioinfo.2017@gmail.com (A.K.); dudtsldl12@naver.com (Y.S.K.); vckaliaku@gmail.com (V.C.K.); 2 Institute of SK-KU Biomaterials, 1 Hwayang-Dong, Seoul 05029, Korea

**Keywords:** *Alcaligenes faecalis*, arylacetonitrilase, catalytic efficiency

## Abstract

Arylacetonitrilase from *Alcaligenes faecalis* ATCC8750 (NitAF) hydrolyzes various arylacetonitriles to the corresponding carboxylic acids. A systematic strategy of amino acid residue screening through sequence alignment, followed by homology modeling and biochemical confirmation was employed to elucidate the determinant of NitAF catalytic efficiency. Substituting Phe-140 in NitAF (wild-type) to Trp did not change the catalytic efficiency toward phenylacetonitrile, an arylacetonitrile. The mutants with nonpolar aliphatic amino acids (Ala, Gly, Leu, or Val) at location 140 had lower activity, and those with charged amino acids (Asp, Glu, or Arg) exhibited nearly no activity for phenylacetonitrile. Molecular modeling showed that the hydrophobic benzene ring at position 140 supports a mechanism in which the thiol group of Cys-163 carries out a nucleophilic attack on a cyanocarbon of the substrate. Characterization of the role of the Phe-140 residue demonstrated the molecular determinant for the efficient formation of arylcarboxylic acids.

## 1. Introduction

Nitrilases are widely distributed in nature and are involved in the transformation of nitriles into ammonia and equivalent carboxylic acids [1]. Nitrilases with diverse substrate specificities have been purified from bacteria [2], fungi, and plants [3,4]. They are useful industrial enzymes that belong to a superfamily [5] that includes amidases, acyltransferases, and N-carbamoyl-amino acid amidohydrolases [6]. The usage of nitrilases seems more attractive over conventional chemical approaches that require harsh conditions, such as high temperatures and the use of concentrated bases or acids [7,8,9].

Various aspects of nitrilases have been reported, including their occurrence, characteristics, applicability, substrate specificity, and mechanisms of action [2,5,10,11,12]. Although some nitrilases have broad substrate specificity, they are largely categorized into three major groups, depending on their specificity toward a substrate, as (i) aromatic nitrilases, (ii) aliphatic nitrilases, or (iii) arylacetonitrilases [2,13]. Therefore, the broad specificity of these nitrilases have indicated their usefulness in the hydrolysis of numerous nitriles [10,12,14].

Although molecular modeling of nitrilases and the interactions between their subunits and substrates have been reported [15,16,17,18], details regarding the molecular determinants of the catalytic efficiency of arylacetonitrilase have never, to the best of our knowledge, been reported. An understanding of the role of amino acid residues at the active microenvironment site may guide future protein design efforts to exploit the ubiquitous and industrially useful enzymes such as nitrilases [19]. Therefore, we present a systematic strategy to identify the molecular determinants underlying the catalytic efficiency of arylacetonitrilase using phenylacetonitrile as a target arylacetonitrile substrate. The approach included multiple sequence alignments and structure-based designs of the active site and involved stepwise site-directed mutagenesis of individual substrate-contacting residues, accompanied by activity screens for variants with modified catalytic activity. We identified a key role of the amino acid at position 140 as being crucial for the catalytic efficiency of *Alcaligenes faecalis* arylacetonitrilase (NitAF). Our findings further demonstrate that the catalytic efficiency of NitAF towards phenylacetonitrile highly depends on the hydrophobicity of the amino acid present at position 140. This residue may be the primary means by which phenylacetonitrile is oriented properly for nucleophilic addition by the thiol group of the cysteine in the catalytic tetrad (Cys–Glu–Glu–Lys) of nitrilase.

## 2. Results

### 2.1. Cloning and Purification of Nitrilase

*A. faecalis* JM3 genomic sequence-related primers were used to acquire nitrilase genes from *A. faecalis* JM3 DNA. The cloned gene contained 1071 bp encoding 357 amino acids. The wild-type and mutant enzymes were purified using a Ni-NTA column to single bands on SDS–PAGE. Enzyme subunits and native molecular mass were approximately 32 and 460 kDa, based on SDS–PAGE and gel filtration chromatography, respectively (Figure S1).

### 2.2. Screening of the Putative Catalytic Residues Using Sequence Alignments

**Figure 1:**
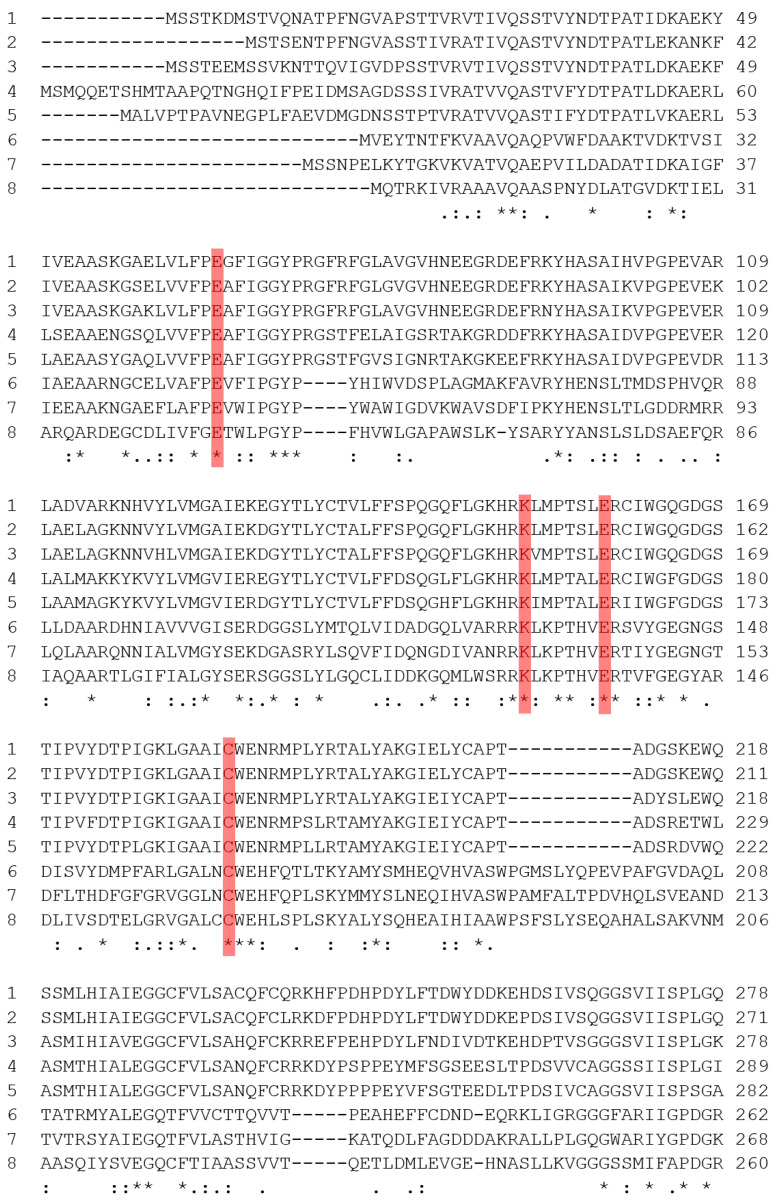
Multiple sequence alignment of nitrilases. 1: NIT1, 2: NIT2, 3: NIT3, and 4: NIT4 of *Arabidopsis thaliana*, 5: NIT4 of *Nicotiana tabacum*, 6: nitrilase of *Rhodococcus rhodochrous* J1, 7: nitrilase of *Rhodococcus rhodochrous* K22, 8: nitrilase of *Alcaligenes faecalis* JM3. The active site residues conserved are shown in the red bar. The symbols “*, :, and .” represent the residues’ perfect identity, high similarity, and low similarity at the site, respectively.

ClustalW was used to align the sequences of arylacetonitrilase from *A. faecalis* JM3, aliphatic nitrilases (EC3.5.5.7) from *Rhodococcus rhodochrous* (J1 and K22), aromatic nitrilases (EC3.5.5.1) from *Arabidopsis thaliana* (NIT1, NIT2, NIT3, and NIT4) and *Nicotiana tabacum* (NIT4), and nitrilase from *Bacillus* sp. (Figure 1). All nitrilases showed complete conservation of the catalytic tetrad Cys–Glu–Glu–Lys (CEEK) [20]. In addition, 79 residues in NitAF were conserved in the sequences of the aligned nitrilases. Given the similar catalytic influence and conformation of conserved residues from active homologous proteins, further screening for the catalytic residues was carried out by homology modeling.

### 2.3. Screening of the Putative Catalytic Residues by Homology Modeling

**Figure 2:**
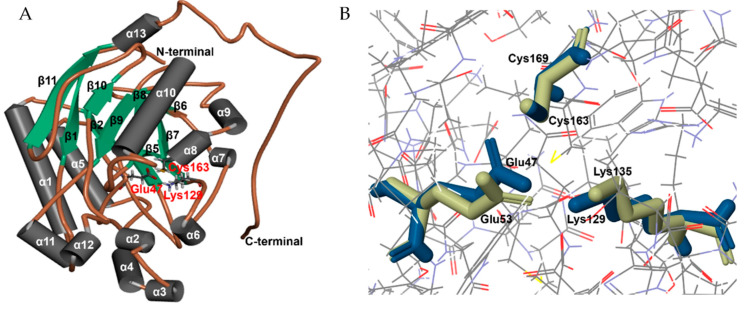
Homology modeling and the catalytic residues of NitAF. (**A**) Complete model of wild-type NitAF showing the catalytic pocket. The crystal structure of the protein Nit6803 from *Syechocystis* sp. PCC6803 was used as a template. (**B**) The catalytic residues of NitAF and Nit6803. The catalytic residues of NitAF (Cys-163, Glu-47, and Lys-129) and Nit6803 (Cys-169, Glu-53, and Lys-135) are similar with regard to position and orientation.

**Figure 3:**
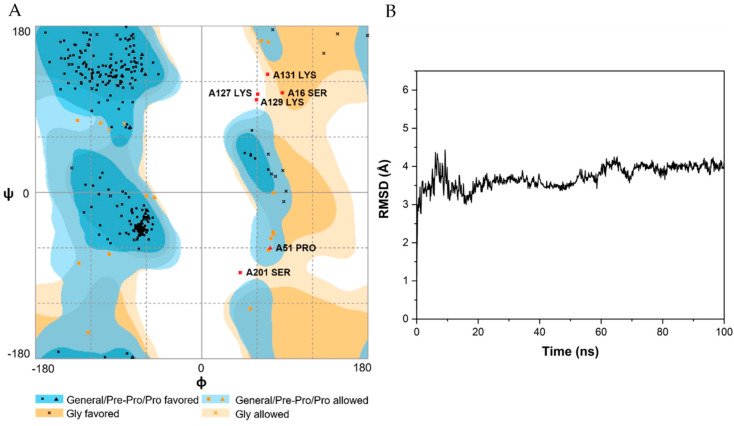
Validation of NitAF model. (**A**) Ramachandran plot of wild-type NitAF. The plot calculation on 3D model of wild-type NitAF was performed with RAMPAGE program. (**B**) MD simulation on wild-type NitAF structure. The RMSD value of the backbone atoms (Cα, N, C) of the wild-type NitAF structure was calculated against the time simulation between 0 and 100 ns.

Homology modeling of wild-type NitAF was performed to better understand the structural basis of the molecular determinants underlying the catalytic efficiency of NitAF and to reduce the number of candidate residues screened (Figure 2A). The NitAF homology model was built using the *Syechocystis* sp. PCC6803 protein (PDB: 3WUY) crystal structure [21] using DS 3.5 software. PROMOTIF analysis of the NitAF model suggested that NitAF contains 11 β-sheets and 13 α-helices (Figure 2A). The orientation of the catalytic residues Cys-163, Glu-47, and Lys-129 of NitAF is similar to that of Cys-169, Glu-53, and Lys-135, respectively, in the *Syechocystis* sp. PCC6803 protein 3WUY (Figure 2B). According to PROCHECK [22], the model derived from the 3WUY template has 84.3%, 11.4%, 1.4%, and 2.9% of residues associated with the favored, allowed, generously disallowed, and disallowed categories, respectively. Similarly, another assessment tool, RAMPAGE [23], suggested that 92.7%, 5.4%, and 1.9% of residues in the derived model were favored, allowed, and outlier regions, respectively (Figure 3A). Approximately 98% of the residues were placed in the combined favored and allowed categories. Therefore, PROCHECK and RAMPAGE validated that the model structure derived from the 3WUY template was of high quality in terms of protein fold. The models were then used in molecular dynamics (MD) simulations to sample conformational changes and the overall structure flexibility. Root mean square deviation (RMSD) was employed to measure the difference between the backbone atoms (Cα, N, C, and O) of protein from its initial structural conformation to its final position. The RMSD of NitAF was calculated for 100 ns simulation to check the stability of the system by the alignment of each snapshots of the MD trajectories with the starting structure during the simulation (Figure 3B). The RMSD increased to approximately 4 Å in 15 ns and then remained stable till the end of the simulation.

**Figure 4:**
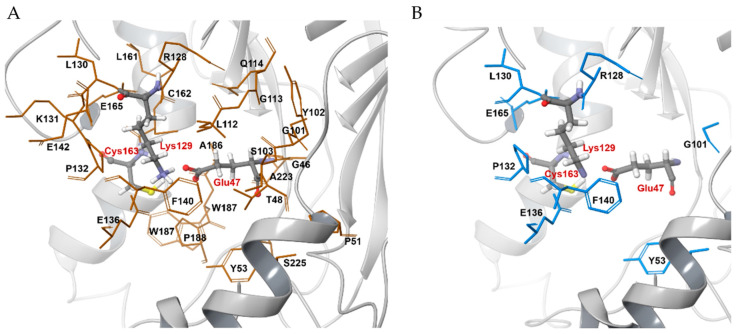
Residues within the 4.5 Å radius centered on the catalytic residues of NitAF. (**A**) In total, 28 residues, including the three conserved catalytic residues (Cys-163, Glu-47, and Lys-129), were found to be within 4.5 Å from the catalytic site of NitAF. (**B**) Conserved residues within the 4.5 Å sphere across the whole nitrilase family. Only 8 out of 25 residues were conserved across the whole nitrilase family.

The substrate phenylacetonitrile was docked into the homology model using DS software. As shown in Figure 4A, 28 residues, including the three highly conserved catalytic residues (Cys-163, Glu-47, and Lys-129), were found within 4.5 Å of the substrate to form the substrate-binding pocket (SBP). Only 8 of these 25 residues were conserved across the whole nitrilase family, including Tyr-53, Gly-101, Arg-128, Leu-130, Pro-132, Glu-136, Phe-140, and Glu-165 (Figure 4B). These residues were selected for analysis as candidate molecular determinants of NitAF catalytic efficiency.

### 2.4. Ala-Substitutions of Putative Residues

To probe their functional roles in catalytic efficiency, selective residues were separately mutated to Ala, and the activity of Ala-substituted phenylacetonitrilase mutants was correlated to that of wild-type NitAF. Substituting all residues except Phe-140 caused almost no alteration in the activity of arylacetonitrilase (data not shown). However, mutant F140A exhibited significantly decreased arylacetonitrilase activity towards phenylacetonitrile, indicating that this residue might be crucial for the enzyme activity of NitAF. The specific activity of the mutant F140A was 1.6 µmol/min/mg-protein towards phenylacetonitrile, corresponding to 9% of the activity of the wild-type enzyme. Although no remarkable activity toward arylacetonitrile was observed after replacing seven residues with smaller amino acids, mutational analysis of Phe-140 indicated that its location in the binding pocket close to the substrate considerably modulated the enzyme’s catalytic efficiency toward arylacetonitrile. This position was recognized as vital for the catalytic efficiency of NitAF for further investigation.

### 2.5. Site-Directed Mutagenesis of Position 140

**Figure 5:**
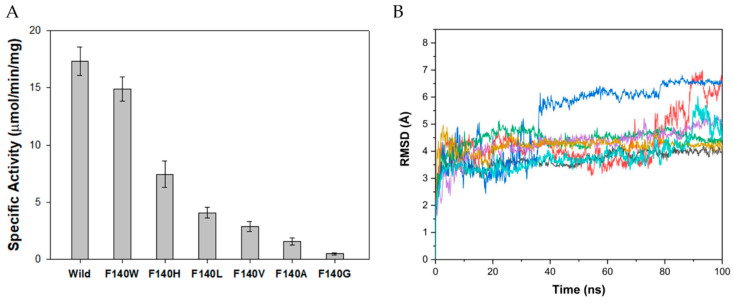
Activity and stability analysis of NitAF variants. (**A**) Catalytic activities of NitAF wild-type and mutants produced by site-directed mutagenesis at position 140. Specific activities with phenylacetonitrile (15 mM) were determined. Each value represents the mean of triplicate measurements with standard deviation less than 15%. (**B**) MD simulation on NitAF variant structures. Wild-type (black), F140G (blue), F140A (red), F140V (light blue), F140L (purple), F140H (green), and F140W (yellow) are shown.

Phe-140 was substituted by nonpolar aliphatic, charged polar, and aromatic residues through a site-directed mutagenesis kit (Stratagene). The mutants were purified (Figure S2), and their catalytic activity for phenylacetonitrile was determined and compared to that of the wild-type enzyme. When Phe-140 was replaced with nonpolar aliphatic amino acids, such as Gly, Ala, Val, and Leu, the activity decreased significantly. The specific activities of F140L, F140V, F140A, and F140G were determined as 4.9, 2.9, 1.6, and 0.5 µmol/min/mg-protein, equivalent to 30%, 17%, 9%, and 3% of the specific activity of wild-type NitAF, respectively (Figure 5A). The activity of mutants containing a nonpolar aliphatic residue was correlated with the size of the residue; thus, a size-dependent correlation was observed between hydrophobicity of the amino acid side chain and enzyme activity. Accordingly, enlarging the side chain of residue 140 near the substrate in the SBP significantly modulated the specific activity of the enzyme due to hydrophobic interactions between the residue at the active site and the substrate. When Phe-140 was substituted by Glu, Arg, and Asp (charged polar amino acids), the mutants displayed no phenylacetonitrilase activity, as these amino acids interrupted the hydrophobic interactions with the substrate. To evaluate the Phe-140 aromatic ring at the active site, the residue was substituted to Trp or His. When Phe-140 was substituted with the aromatic residue Trp, the F140W mutant had an activity similar to that of the wild-type, suggesting that the Phe and Trp aromatic ring was involved in binding and/or immobilizing the benzene ring of phenylacetonitrile. However, the F140H mutant exhibited a decreased activity, probably because of the hydrophilic group in the His side chain (Figure 5A). MD simulation was carried out on the mutants (F140A, F140G, F140H, F140L, F140W, and F140V) to estimate conformational changes and the overall structure flexibility. The RMSD values of the mutant structures were calculated for 100 ns simulation. The average RMSD values of the mutants F140A, F140G, F140H, F140L, F140W, and F140V were 4.4 Å, 5.3 Å, 4.4 Å, 4.3 Å, and 3.8 Å, respectively, which were larger than that of the wild-type (3.7 Å) during the MD simulation (Figure 5B).

### 2.6. Kinetic Analysis of the Wild-Type and Mutant Enzymes

For further evaluation, purified (wild-type or mutant) enzyme kinetic parameters for phenylacetonitrile were determined (Table 1). The kinetic parameters of the mutant enzymes substituted by Arg, Glu, or Asp were not determined due to their low specific activities (below 1% compared to the wild-type). Mutants with nonpolar aliphatic residue catalytic efficiency (*k_cat_*/*K*_m_) were directly associated with decreases in amino acid side chain size. For the F140G mutant, the low activity was ascribed to lower *k_cat_* (8.60 s^−1^) and higher *K*_m_ (24.1 mM) than those of the wild-type. The F140W mutant exhibited a slightly higher *k_cat_* value than the wild-type enzyme.

**Figure 6:**
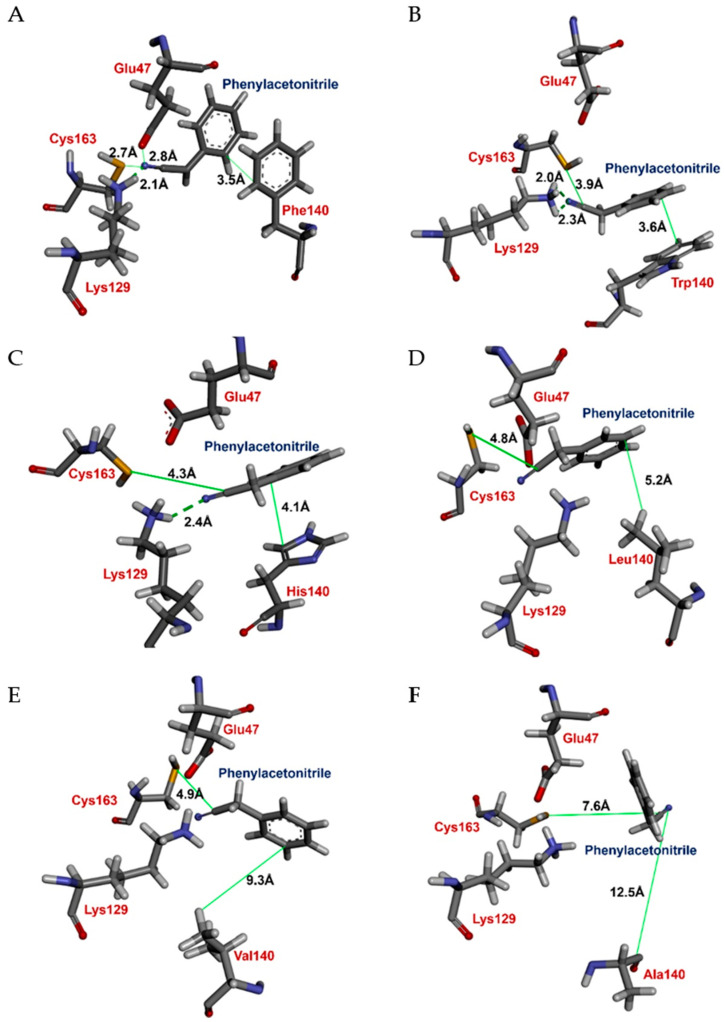
Model image of the NitAF active site. Phenylacetonitrile was docked into the substrate-binding pocket (SBP) of the (**A**) wild-type NitAF, (**B**) F140W, (**C**) F140H, (**D**) F140L, (**E**) F140V, and (**F**) F140A mutants. The distances between Cys-163 and phenylacetonitrile in NitAF and the F140A mutant were 2.7 Å and 7.6 Å, respectively. The green dotted line represents the H-bond, and the green straight line indicates the distance between the substrate and neighbor amino acid residues. Amino acids are shown as stick models.

According to the kinetic constants of the mutants, Δ(ΔG) values were determined (Table 1). To examine the interactions of the substrate with every amino acid residue that mutated in the NitAF model, the distance between residues and phenylacetonitrile was assessed using the predicted model constructed by MD simulation (Figure 6). In contrast to NitAF (wild-type), the F140A and F140G mutants showed significantly lower catalytic efficiencies of 3.71 and 0.36 s^−1^ mM^−1^, respectively, and higher Δ(ΔG) values of 9.50 and 15.2 kJ mol^−1^, respectively, possibly due to the increased distance between the residue and substrate (Figure 6F). On the other hand, the mutant F140W (Figure 6B) did not show significant change in catalytic efficiency.

## 3. Discussion

Nitrilases, as thiol enzymes, form covalent thioimidate complexes by attacking the carbon of nitriles (R–CN). The catalytic tetrad of nitrilase consists of CEEK; the cysteine carries out a nucleophilic attack on the cyanocarbon, the glutamate functions by mediating the transfer of a proton as a general base, and the lysine plays an essential role in the stabilization of a tetrahedral intermediate [20,21,24]. Although previous investigations have aimed to elucidate the role of an amino acid residue in the substrate specificity of nitrilases using mutational and computational approaches, its role in the catalytic efficiency has not been reported; thus, a comprehensive understanding of the factors contributing to the catalytic efficiency of nitrilases remains elusive. To identify the molecular determinant for the catalytic efficiency of NitAF, we carried out a systematic strategy of screening for conserved residues across the whole nitrilase family through alignments of multiple sequences, and we carried out MD simulations to identify conserved residues contacting the phenylacetonitrile substrate, followed by site-directed mutagenesis.

When phenylacetonitrile, an arylacetonitrile substrate, was docked into the NitAF (wild-type) active site, a smaller distance was observed between the substrate cyanocarbon to Cys-163 sulfur (2.7 Å; Figure 5A) than for the F140A mutant (7.6 Å; Figure 5F). This increased distance in the case of the F140A (Figure 5F) mutant seems to explain the lower *k_cat_* value of the F140A mutant for the substrate. Moreover, the catalytic efficiency of the mutants containing nonpolar aliphatic residues was F140L > F140V > F140A > F140G, which was linked to a decrease in the size of the side chain (Table 1). Mutations that resulted in a smaller residue at position 140 are anticipated to expand the SBP, which might cause low substrate-binding affinity. In addition, the removal of the large phenyl group of F140 appeared to prevent the proper orientation of the substrate, phenylacetonitrile, for nucleophilic attack by the thiol group of Cys-163 (Figure 5B). Consequently, the decrease of phenylacetonitrilase activity for mutants with nonpolar aliphatic residues indicated the importance of the aromatic ring residue at position 140.

Moreover, aromatic–aromatic interactions are ubiquitous [25] and have been suggested to be stabilizing forces in globular protein structures [26], as the interactions may stabilize protein–ligand complexes and folded proteins [27,28]. In this study, our post docking assessment indicated that the side-chain of Phe-140 provided similar stabilizing protein–ligand complexes with the aromatic ring of phenylacetonitrile (Figure 5A). Indeed, the Δ(ΔG) value of the wild-type NitAF was much lower than that of other mutant NitAFs (Table 1), probably due to the stabilization of the protein–substrate complex through the aromatic–aromatic interaction. Analysis of docked protein–ligand complexes showed that the distance between geometric centroids of the aromatic pair was 5.3 Å, while the distance of the closest approach of ring carbon atoms was 3.5 Å (Figure 5A). This is within the range of previously reported aromatic–aromatic interaction distances [26].

Although aromatic–aromatic interactions have been studied as recognition factors, aromatic groups may interact in one of several geometries, depending on the aromatic rings involved [25], including edge to face, offset stacked, and face to face orientations. However, we found that the aromatic ring arrangement of Phe-140 and phenylacetonitrile in the wild-type NitAF was similar to the offset stacked orientation (Figure 5A). This is one of the important geometric arrangements of aromatic rings. The offset stacked geometry increases van der Waals forces and hydrophobic interactions with a more buried surface area. It is likely that these results explain how Phe-140 provides stabilized stacking interactions to phenylacetonitrile via the functional group of the substrate oriented toward the CEK catalytic pocket. On the other hand, when Phe-140 was mutated to Ala and Gly, there was a significant decrease in enzyme activity, likely due to the absence of an aromatic side-chain, resulting in the loss of stacking interactions in the protein–ligand complex. As a result, phenylacetonitrile loosely bound the CEK catalytic pocket, and the orientation of the functional group protruded outward from it, resulting in the loss of enzyme activity (Figure 5F).

## 4. Materials and Methods

### 4.1. Chemicals and Materials

Chemicals and the assay kit were procured from Sigma-Aldrich (St. Louis, MO, USA). Enzymes related to molecular analysis were obtained from New England Biolabs (Beverly, MA, USA). Kits for plasmid preparation, the purification of PCR products, and gel extraction were purchased from Qiagen (Valencia, CA, USA). Primers were procured from Bioneer (Seoul, Korea). Biochemicals were supplied by Roche Diagnostics (Madison, WI, USA). The pTrc99A vector was purchased from Amersham Pharmacia Biotech. pTrcHis_6_ was derived from the pTrc99A vector to prepare a C-terminal His_6_ tag.

### 4.2. Bacterial Strains and Growth Conditions

Arylacetonitrilase source *A. faecalis* JM3 ATCC 8750 was grown on nutrient agar at 37 °C. *Escherichia coli* BL-21 and DH5α were obtained from Novagen (Darmstadt, Germany). To express the protein, the recombinant *E. coli* was cultured in medium (Luria-Bertani, LB) containing ampicillin (100 μg/mL) at 37 °C with agitation at 250 rpm until the bacterial culture attained an OD_600_ of 0.6. Further, 0.4 mM of isopropyl-β-d-thiogalactopyranoside (IPTG) was added to the medium, followed by incubation at 25 °C with agitation at 150 rpm for 8 h [29].

### 4.3. Construction of the pTrc-nitAF Plasmid

Amplification of the arylacetonitrilase gene was performed through PCR using the *A. faecalis* JM3 genomic DNA as the template. For cloning, primers were constructed from the *A. faecalis* JM3 nitrilase gene (GenBank accession number P20960). Primers (Forward (5′-GGGAATTCATGCAGACAAGAAAAATCG) and reverse (5′-ATAAAGCTTGTGGTGGTGGTGGTGGTGGGACGGTTCTTGCACCAGTAGCG)) were labeled as underlined restriction positions for *EcoR*I and *Hind*III, respectively. The DNA fragment was refined from the PCR purification kit by Promega (Madison, WI, USA). The purified product was cloned into the suggested restriction positions (*EcoR*I and *Hind*III) of pTrc-99A to yield the pTrc-nitAF construct for transformation into *E. coli* BL21 (DE3).

### 4.4. Site-Directed Mutagenesis of pTrc-nitAF

Mutagenesis was performed using a QuikChange kit (Stratagene, La Jolla, CA, USA) using pTrc-nitAF plasmid DNA. The positive mutant gene plasmids were transformed into *E. coli* BL21 and ampicillin-resistant colonies were utilized for protein expression. Mutants of pTrc-nitAF were expressed according to the same procedure for the wild-type enzyme, as described below.

### 4.5. Arylacetonitrilase Expression and Purification

The pTrc-nitAF-transformed, overnight-grown BL21 (DE3) cells (Invitrogen, Carlsbad, CA, USA) were diluted 200-fold in LB with ampicillin (100 µg/mL) and grown at 30 °C until the suspension attained an OD_600_ of ~0.6. IPTG (0.1 mM) was used to induce protein expression for 3 h of incubation. Prior to cell disruption through ultrasonication (Fisher Scientific, Pittsburgh, PA, USA), cells were treated with lysozyme (1 mg/mL) in potassium phosphate buffer (50 mM) containing 0.1 mM each of dithiothreitol, EDTA, and phenylmethyl-sulfonyl fluoride as protease inhibitors. After removing cell debris, the supernatant loaded into the Ni-NTA column (3.4 × 13.5 cm, QIAGEN) was pre-equilibrated using lysis buffer (NaH_2_PO_4_, 50 mM; NaCl, 300 mM; pH 8). Further, free proteins were separated using washing buffer (NaH_2_PO_4_, 50 mM; NaCl, 300 mM; imidazole, 20 mM; pH 8). The bound arylacetonitrilase was recovered using elution buffer (NaH_2_PO_4_, 50 mM, NaCl, 300 mM; imidazole, 250 mM; pH 8). Similarly, recombinant nitrilases (tagged with histidine residues at the C terminal) were purified using the Ni-NTA system. Protein quantification and purity confirmation were evaluated by Bradford [30] and sodium dodecyl sulfate–polyacrylamide gel electrophoresis (SDS–PAGE) methods, respectively. 

### 4.6. Kinetic Analysis of NitAF and Mutants

Arylacetonitrilase activity was analyzed by measuring evolved ammonia as per a previously reported method [31,32,33]. Phenylacetonitrile (0.5–30 mM) was used as a substrate in a buffer (50 mM, pH 7.5) with methanol (10%) and the enzyme at 30 °C for 30 min. Thereafter, 20 mM of HCl was added to stop the reaction. Nitrilases kinetic parameters were calculated by the Michaelis–Menten method. Enzyme activity (U) was determined as the amount of enzyme required to produce 1 µmol of product per minute under standard assay conditions at 30 °C.

### 4.7. Protein Electrophoresis

Electrophoresis (SDS–PAGE, Bio-Rad, Richmond, CA, USA) was conducted using polyacrylamide gels (12%). The BCA method was used for protein quantification using bovine serum albumin as the standard. Prior to the SDS–PAGE, samples were incubated at 100 °C for 5 min. Standards of various molecular weights were procured from Bio-Rad.

### 4.8. Homology Modeling

The models for NitAF and the mutant (F140A and F140G) proteins were constructed. The 3D structure of NitAF was created by Modeler in Discovery Studio 3.5 (DS 3.5, Accelrys Software Inc., San Diego, CA, USA). BLAST analysis using the protein database (PDB) revealed a considerable sequence identity (36%, 110/303) between NitAF and the protein Nit6803 (PDB accession code 3WUY). The crystal structure of the protein Nit6803 (resolution, 3.1 Å) [21] from *Syechocystis* sp. PCC6803 was employed (as a template) to generate a comparative modal structure by fulfilling criteria based on spatial restraints and geometry. To verify the model, the Profile-3D score was calculated using the Profile-3D module in DS. The Profile-3D score was found to be 127, compared to a total score of 145. The built model was validated through the superimposition of the template, and RMSD was found to be 0.88 Å for the carbon alpha atoms. The created structure was enhanced through refining loop conformational (amino acid sequence) compatibility to established PDB structures by the Protein Health module in DS. All the studies related to simulation were performed by HP XW6200 Workstation (Intel Xeon 3.2 GHz processor).

Docking analysis was performed by employing a built protein model after the addition of hydrogen atoms that were minimized for stable energy and relaxed conformations with close contacts. The NitAF active site comprised specified amino acid residues covered inside the sphere radius of 4.5 Å towards the catalytic residues (Cys-163, Glu-47, and Lys-129) at the center. The C-DOCKER module in DS was employed to dock phenylacetonitrile into the arylacetonitrilase CEK-binding pocket using a previously described method [34]. High-temperature MD was used to generate random ligand conformations. Furthermore, candidate poses were generated through random rotations (rigid-body) and simulated annealing. The energy minimization of the protein structure and substrate along with their complexes was performed using the CHARMm forcefield function in DS to refine the ligand poses [35,36]. C-DOCKER energy-based docked substrate conformation was recovered for post-docking assessment, and substrate orientation (lowest interaction energy) was selected for the next set of docking.

Mutant NitAF models were generated by substituting a single residue at position 140 of the wild-type NitAF model using the generated mutant DS protocol. The build protocol mutated chosen residues and optimized their conformation and any adjacent residues that were present within a specified cutoff radius. Then, phenylacetonitrile, an arylacetonitrile substrate, was docked into the active site of the mutant NitAF models.

### 4.9. MD Simulation

MD simulation was performed for 100 ns to simulate a structural motion of the modeled NitAF via DS. Initially, the system was filled with TIP3P water molecules in an orthorhombic box. The enzyme was placed 10 Å from the boundary of the box, and the periodic boundary condition was maintained during the simulation. Counter-ions, such as sodium and chloride, were included to neutralize the system. The Standard Dynamics Cascade protocol in DS was utilized to perform the simulation as follows: The steepest descent algorithm was used for the first step energy minimization for 1000 steps to relax the system and remove the extra clashes within the atoms of the enzyme. Furthermore, the minimized system was subjected to the second stage energy minimization for 2000 steps with a conjugate gradient method to obtain the minimum energy initial structure. In the third step, the system temperature was increased from 50 to 300 K for 2 fs with a harmonic restraint on backbone atoms. Additionally, equilibration was conducted at a later stage to allow water molecules to diffuse into the system at the desired temperature. Finally, production was performed for 100 ns at 300 K and at a constant pressure of 1 atm. The snapshots of the production stage were saved at every 2-ps interval

### 4.10. Analysis Methods and Database

Absorbance of the samples was noted spectrophotometrically (Varian Cary 100 Bio UV–Vis spectrophotometer, Palo Alto, CA, USA) [37]. BLAST analysis was performed using *nitAF* gene sequences of *A. faecalis* JM3 to compare with associated enzymes, and multiple sequence alignments between the *A. faecalis* JM3 NitAF and related enzymes were conducted using ClustalW (https://www.ncbi.nlm.nih.gov/).

## 5. Conclusions

In the present study, we showed that the activity of NitAF, an arylacetonitrilase, can be controlled by rational design of the SBP residues. Residue 140 of NitAF was found to be a molecular determinant modulating the catalytic efficiency of the enzymes. That is, hydrophobicity of the residue at position 140 determines the catalytic efficiency of NitAF. This is the first study reporting the molecular determinants for the catalytic efficiency of arylacetonitrilase. Evaluation of the structure of NitAF using cryogenic electron microscopy or X-ray crystallography is required to validate the homology models and structural interpretations suggested above. These findings should provide further insights on arylacetonitrile oxidation and will be useful for research on arylacetonitrilase for the production of industrially valuable arylcarboxylic acid compounds.

## Figures and Tables

**Table 1:** Kinetic parameters of wild-type NitAF and mutants for hydrolyzing phenylacetonitrile.

Enzyme	*K*_m_ (mM)	*k*_cat_ (s^−1^)	*k*_cat_/*K*_m_ (s^−1^ mM^−1^)	Δ(ΔG) ^a^ (kJ mol^−1^)
Wild-type	0.86 ± 0.07	142 ± 10	165	0
F140W	1.45 ± 0.12	143 ± 12	98.6	1.30
F140H	0.89 ± 0.10	63.5 ± 4.7	71.4	2.11
F140L	2.13 ± 0.21	38.8 ± 3.8	18.2	5.55
F140V	2.95 ± 0.32	25.0 ± 2.4	8.48	7.48
F140A	4.20 ± 0.41	15.6 ± 1.2	3.71	9.50
F140G	24.1 ± 2.2	8.60 ± 0.07	0.36	15.2

^a^ Δ(ΔG) = −*RT* ln[(*k*_cat_/*K_m_*)mut/(*k*_cat_/*K_m_*)wt], where *R* is the ideal gas constant, *T* is the temperature in Kelvin, mut is mutant, and wt is wild-type. F140D, F140E, F140R enzyme: not determined due to no or low activity.

## Supplementary Materials

Supplementary materials can be found at https://www.mdpi.com/1422-0067/21/21/7859/s1. Figure S1: Determination of the molecular mass of *Alcaligenes faecalis* NitAF by SDS-PAGE and gel filtration chromatography; Figure S2: Purification of NitAF and its mutant nitrilases at position 140 by Ni-NTA chromatography (Coomassie-stained 12% SDS-PAGE gel).
