# Supplementary material for: Phe-140 Determines the Catalytic Efficiency of Arylacetonitrilase from Alcaligenes faecalis
Source: Int J Mol Sci. 2020 Oct 23;21(21):7859. doi: 10.3390/ijms21217859 (PMC7660301; doi:10.3390/ijms21217859)
Supplement: Supplementary file 1 [file ijms-21-07859-s001.pdf]

International Journal of
Molecular Sciences

MDPI

Supplementary file

Phe-140 Determines the Catalytic Efficiency of Arylacetonitrilase from *Alcaligenes faecalis*

Jung-Soo Kim [1,†], Sanjay K. S. Patel [1,2,†], Manish K. Tiwari [1,†], Chunfen Lai [1], Anurag Kumar [1], Young Sin Kim [1], Vipin Chandra Kalia [1] and Jung-Kul Lee [1,2,*]

1 Department of Chemical Engineering, Konkuk University, Seoul 05029, Korea; junnie05@hanmail.net (J.-S.K.); sanjaykspatel@gmail.com (S.K.S.P.); manish.bme@gmail.com (M.K.T.); cfenlai01@gmail.com (C.L.); anuragbioinfo.2017@gmail.com (A.K.); dudtsldl12@naver.com (Y.S.K.); vckaliaku@gmail.com (V.C.K.)

2 Institute of SK-KU Biomaterials, 1 Hwayang-Dong, Seoul 05029, Korea

* Correspondence: jkrhee@konkuk.ac.kr; Tel: +82-2-450-3505

† These authors equally contributed to this work.

Received: 16 August 2020; Accepted: 20 October 2020; Published: 23 October 2020

(A)

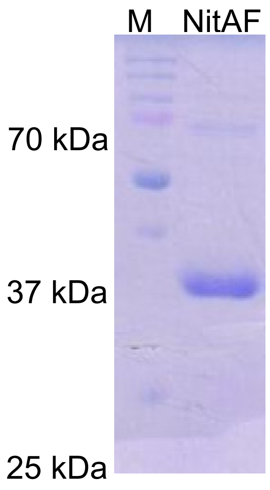

(B)

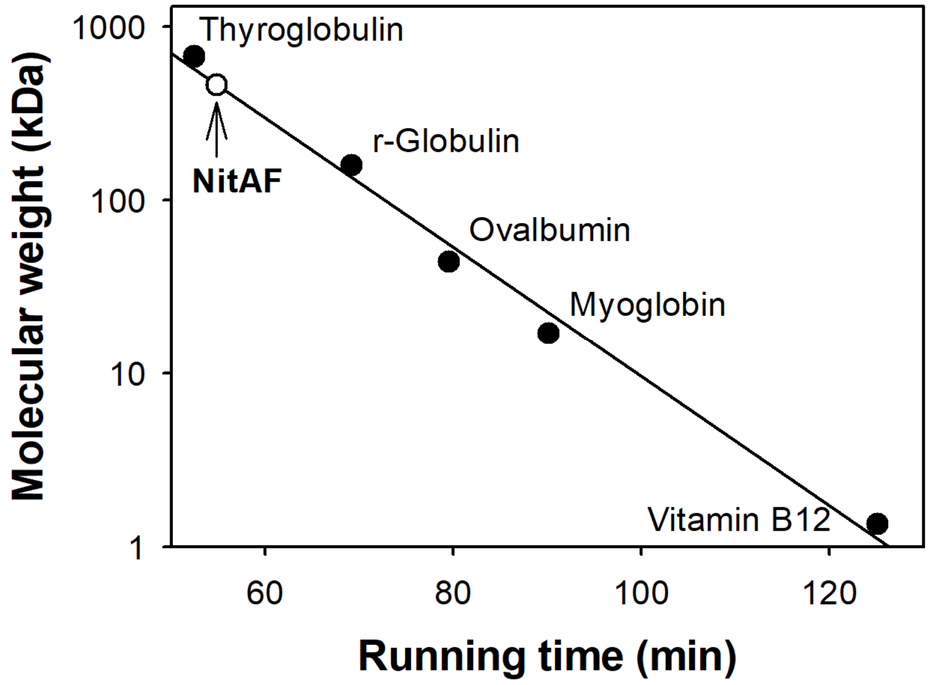

Figure S1. Determination of the molecular mass of *Alcaligenes faecalis* NitAF by SDS-PAGE and gel filtration chromatography. (**A**) Purification of *Alcaligenes faecalis* nitrilase (NitAF) by Ni-NTA chromatography (Coomassie-stained 12% SDS-PAGE gel). (**B**) Native molecular weight of the enzyme estimated by gel filtration to be 460 kDa. The column was calibrated with standard molecular weight proteins such as thyroglobulin (669 kDa), γ-globulin (158 kDa), ovalbumin (44 kDa), myoglobin (MW 17 kDa), and vitamin B12 (13.5 kDa). The gel filtration standard proteins were purchased from Bio-Rad (Hercules, CA, USA).

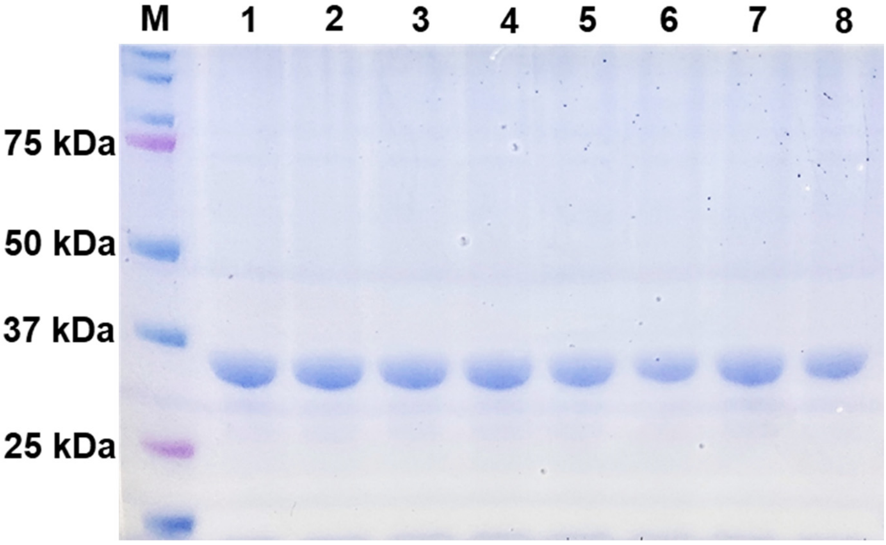

Figure S2. Purification of NitAF and its mutant nitrilases at position 140 by Ni-NTA chromatography (Coomassie-stained 12% SDS-PAGE gel). M, Molecular marker; 1, wild-type NitAF; 2, F140D; 3, F140W; 4, F140H; 5, F140L; 6, F140V; 7, F140A; 8, F140G.
